# Psychosocial barriers and facilitators for adherence to a healthy lifestyle among patients with chronic kidney disease: a focus group study

**DOI:** 10.1186/s12882-022-02837-0

**Published:** 2022-06-11

**Authors:** Cinderella K. Cardol, Karin Boslooper-Meulenbelt, Henriët van Middendorp, Yvette Meuleman, Andrea W. M. Evers, Sandra van Dijk

**Affiliations:** 1 Health, Medical and Neuropsychology Unit, Leiden University, Leiden, The Netherlands; 2 Department of Internal Medicine, University Medical Center Groningen, University of Groningen, Groningen, The Netherlands; 3 Department of Clinical Epidemiology, Leiden University Medical Center, Leiden, The Netherlands; 4 Medical Delta, Leiden University, TU Delft, and Erasmus University Rotterdam, Delft, The Netherlands

**Keywords:** Lifestyle adherence, Psychosocial determinants, Self-management interventions, Chronic kidney disease (CKD), Qualitative research, Focus groups, Thematic analysis, Theoretical Domains Framework (TDF)

## Abstract

**Background:**

Progression of chronic kidney disease (CKD) may be delayed if patients engage in healthy lifestyle behaviors. However, lifestyle adherence is very difficult and may be influenced by problems in psychosocial functioning. This qualitative study was performed to gain insights into psychosocial barriers and facilitators for lifestyle adherence among patients with CKD not receiving dialysis.

**Methods:**

Eight semi-structured focus groups were conducted with a purposive sample of 24 patients and 23 health care professionals from four Dutch medical centers. Transcripts were analyzed using thematic analysis. Subsequently, the codes from the inductive analysis were deductively mapped onto the Theoretical Domains Framework (TDF).

**Results:**

Many psychosocial barriers and facilitators for engagement in a healthy lifestyle were brought forward, such as patients’ knowledge and intrinsic motivation, emotional wellbeing and psychological distress, optimism, and disease acceptance. The findings of the inductive analysis matched all fourteen domains of the TDF. The most prominent domains were ‘social influences’’and ‘environmental context and resources’, reflecting how patients’ environments hinder or support engagement in a healthy lifestyle.

**Conclusions:**

The results indicate a need for tailored behavioral lifestyle interventions to support disease self-management. The TDF domains can guide development of adequate strategies to identify and target individually experienced psychosocial barriers and facilitators.

**Supplementary Information:**

The online version contains supplementary material available at 10.1186/s12882-022-02837-0.

## Background

For patients in the non-dialysis-dependent stages of chronic kidney disease (CKD), engaging in a healthy lifestyle is crucial, as it can postpone further loss of kidney function and prevent cardiovascular complications [1]. Key lifestyle behaviors in CKD include engaging in regular physical activity, refraining from smoking, maintaining a healthy weight, and adhering to dietary regimens and medication prescriptions [2]. Unfortunately, engaging in healthy lifestyle habits is difficult for most patients. A large observational cohort study showed that only 2% of the patients with mild-to-moderate CKD achieved all four lifestyle recommendations assessed [1]. Almost a quarter of the patients were regular smokers, nearly half of the cohort reported limited physical activity, approximately 80% did not meet dietary regimens, and a similar percentage were overweight or obese [1].

One of the possible explanations for this non-adherence to a healthy lifestyle is that modifying lifestyle is not the only challenge that patients face. The integration of kidney disease and its medical management into daily life requires extensive coping skills, such as accepting the diagnosis and prognosis, as well as coping with physical symptoms and social implications of CKD [3]. Consequently, many patients experience diminished psychosocial functioning, even patients in early stages of CKD [4–6]. For example, recent studies showed that one-quarter to a third of patients with CKD not receiving dialysis are affected by psychological distress, that is, symptoms of depression or anxiety–such as sadness, loss of interest, irritability, nervousness, or restlessness [4, 5].

Identification and treatment of psychosocial problems is important, since psychosocial functioning determines how patients cope with chronic disease and their ability to change lifestyle habits [7]. Psychosocial influences on self-management and lifestyle behaviors can range from internal (e.g., behaviors, cognitions, and emotions) to coping with external (i.e., social and environmental) determinants. For instance, depression and anxiety symptoms and a lack of social support have been associated with medication non-adherence in kidney transplant recipients [8]. Such knowledge on psychosocial factors that hinder or facilitate the engagement in healthy lifestyle behaviors is imperative for the development of effective lifestyle interventions to aid patients with CKD. Yet, relatively few studies have explored the barriers and facilitators for successful adherence to lifestyle guidelines in this population. Also, the existing literature predominantly focused on adherence to dietary regimens, mainly among patients treated with hemodialysis [9–11]. As the dialysis treatment for patients with kidney failure is more burdensome and requires different lifestyle adaptations (e.g., stringent fluid restrictions) compared to disease management for patients with CKD not receiving dialysis, barriers and facilitators may also differ between the two CKD populations [2]. On the contrary, except for a strict adherence to immunosuppressive medications after kidney transplantation, most general lifestyle recommendations for kidney transplant recipients and other patients with CKD not receiving dialysis are similar, especially after the postoperative recovery period [2, 12, 13]. Also, the lifestyle measures have similar purposes for both groups, that is, to delay disease progression and to lower cardiovascular risk [2, 12, 13].

Most studies among patients with CKD did not use a theoretical framework such as the Theoretical Domains Framework (TDF, [14, 15]). This framework synthesizes a number of behavior change theories into 14 domains that determine behavior, such as skills, reinforcement, social influences, and emotion. The TDF has been used in qualitative studies among populations with other chronic diseases, including those that address lifestyle change [15, 16]. The TDF may be helpful to disentangle and structure barriers and facilitators, and importantly, the TDF domains can be translated to evidence-based intervention strategies and behavior change techniques (BCTs) to address barriers and promote the desired lifestyle behaviors [14, 15]. Last, to our knowledge, few studies included the perspectives of health professionals. It is important to explore the barriers and facilitators for lifestyle adherence that health professionals observe among their patients, since they experience what works for whom. For successful implementation of lifestyle interventions in health care settings, health professionals should find them beneficial to their daily practice [17]. When exploring both patients and health professionals’ perspectives, similarities and differences can be revealed and incorporated into an intervention design [9].

This study entails a further exploration of factors that are related to the key lifestyle behaviors in CKD: keeping a healthy diet and weight, engaging in regular physical activity, refraining from smoking, and adhering to medication prescriptions [2]. To gain in-depth insight into patients and health professionals’ perspectives on psychosocial influences on adherence to a healthy lifestyle, a semi-structured focus group study was conducted among patients and health professionals and data were mapped onto the TDF. This study had two aims:﻿  (1) to identify psychosocial barriers and facilitators for engaging in a healthy lifestyle among patients with CKD not receiving dialysis, and  (2) to explore which intervention strategies are needed to address such barriers and facilitators.

## Methods

### Setting

This focus group study is part of the E-health Guidance in identifying and Overcoming psychological barriers for Adopting a healthy Lifestyle among patients with chronic kidney disease (E-GOAL) study (Netherlands Trial Registry, study number: NL7338), which entails the development and evaluation of a self-management electronic health intervention. In line with the exploratory nature of the study, focus groups instead of individual interviews were conducted, as participant interaction could create a chain of thoughts and ideas and the group dynamics may provide a breadth of perspectives and information [18]. Four focus groups with patients and four with health professionals took place between August 2017 and February 2018 in four medical centers distributed throughout The Netherlands, of which three university medical centers and one non-academic center. The study was approved by the Medical Research Ethics Committee Leiden The Hague Delft (MREC LDD P17.090) and was performed in accordance with the 1964 Helsinki declaration and its later amendments. The COnsolidated criteria for REporting Qualitative research (COREQ, [19]) were followed (Additional File 1).

### Participant selection and recruitment

We used purposive sampling to include a heterogeneous sample in order to explore a wide range of perspectives [20]. For this purpose, health professionals were asked to recruit Dutch-speaking patients of 18 years or older, with spread in sociodemographic characteristics (i.e., in age and gender), different non-dialysis-dependent CKD stages (including kidney transplant recipients > 1 year ago), and diverse experiences with adapting lifestyle behaviors (i.e., regarding different lifestyle domains, level of difficulty to adhere to a healthy lifestyle, and amount of professional support received). Patients were invited to participate by their nephrologist or nurse practitioner during hospital visits. To gain insights from different occupational perspectives, health professionals of all relevant occupations in CKD care (e.g., nephrologists, nurse practitioners, and dieticians) were invited to participate via email by a nephrologist from the research team who worked at the participating departments. Participation was voluntary and without compensation, except for reimbursement of patients’ travel expenses (full compensation of public transport or mileage allowance). Participants received verbal and written information regarding study purposes and procedures and provided written informed consent prior to participation.

Participants were recruited until six to ten individuals for each focus group were scheduled at a convenient date and time to maximize attendance. Twelve patients were eventually unable to attend (for nine of them the scheduled date was inconvenient, two patients cancelled due to health reasons, and one patient cancelled due to personal circumstances). Five health professionals were unable to attend on the scheduled date due to work-related obligations. Focus groups were held until data saturation (i.e., until no new themes were brought forward). In case of last-minute cancellations, it was decided to proceed with a focus group if at least four participants were present, to maintain sufficient opportunity for group discussion.

### Data collection and content

The focus group sessions lasted between 1.5 and 2.5 h and were moderated by the first author, a female PhD candidate in medical psychology, who had received training in conducting and analyzing focus group discussions. The author had limited interactions by email or phone with participants before the focus group sessions, except for four participating nephrologists, with whom she already had a professional relationship. The participants were informed that the moderator was a researcher working on a lifestyle program to support patients with CKD. An instructed observer (female) took field notes on group dynamics and nonverbal communication. The sessions were audio recorded with permission of the participants.

A semi-structured focus group question guide was developed in accordance with the project aims and literature guidelines [20, 21], partly based on a previous study of the research group [9]. The focus group guide was refined in collaboration with a patient with CKD, who provided feedback on question structure, interpretation, and comprehensibility of wording. Open-ended questions were included in the semi-structured focus groups, allowing the exploration of themes as they arose. The moderator probed responses and stimulated in-depth discussions and engagement of all participants. Participants answered questions about 1) the perceived consequences and difficulties to adjust to CKD, 2) experiences regarding the adherence to a healthy lifestyle. This included the perceived barriers and facilitators in general and for each of the specific lifestyle recommendations, 3) the role of psychosocial barriers and facilitators and, specifically, psychological distress, and 4) their ideas about how to target psychosocial barriers and facilitators in a support program. To introduce our assumption that psychosocial issues and psychological distress may play an important role, after an open discussion of all themes, two translated quotations about the psychological impact of chronic disease from qualitative studies among other chronically ill populations were shown, and participants were invited to discuss whether they recognized these. A summary of the focus group guide is shown in Table A1 (Additional File 2). By the end of the sessions, participants individually wrote down a top-3 of barriers and facilitators they considered most important. Furthermore, they completed a short questionnaire on sociodemographic characteristics.

### Data analysis

The focus group moderator and observer discussed the main themes in a debriefing directly after each focus group. Suiting the exploratory purpose of the study, the transcripts were analyzed using a thematic analysis approach. Analysis was conducted following the six phases outlined by Braun and Clarke [22], and with use of Atlas.ti version 7.5.6 software. In the first phase, the first author transcribed the sessions verbatim, based on recordings and field notes. The author reviewed all transcripts and marked first ideas for codes. Unclear statements were clarified by contacting the concerning participants, to ensure that their perspectives were adequately represented. Phase 2 comprised inductively coding the transcripts by categorizing all relevant data under codes. Phase 3 involved combining different codes into themes. In phase 4, the themes were reviewed and then deductively classified into the 14 domains of the TDF (v2, [14, 15]). The TDF entails a synthesis of 14 main behavior change determinants from key theories, e.g. knowledge, intentions, environmental context and resources, and behavioral regulation [14]. The TDF is validated for use in behavior change research [15], and has been used to understand determinants of behavior change among patients with CKD [16, 23]. The TDF was used because the themes fitted its domains well and the framework was helpful to disentangle and structure the findings. In phase 5, the TDF domain definitions were used to describe the content and refine the themes within each domain. Last, in the sixth phase, the current article was written. In this phase, similarities and differences between patients and health professionals’ focus groups were taken into account in two ways: First, the Atlas.ti software was used to mark to which focus group the data within each domain and theme belonged, which made differences and parallels between the two participant groups visible. Second, the top-3 barriers and facilitators considered most important by each participant were analyzed: Three points were given to a theme a participant found most important, two to their second, and one to their third choice. Then, all top-3 barriers and facilitators were categorized into the TDF domains. Percentages of the points given to each domain were calculated for the full sample, and also per participant category. Finally, to structure the results section of the report, an existing categorization of the fourteen TDF domains was used [15], into three overarching components that are considered essential for behavior and behavior change to occur: Capability, Opportunity, and Motivation (the ‘COM-B’ system, [14]).

In both the inductive and deductive coding stages, triangulation across three investigators—all experienced in qualitative data analysis—repeatedly took place. The first author analyzed all transcripts in accordance with the six phases, from transcription to manuscript drafting. The second author (a female physician researcher and PhD candidate in nephrology) and the last author (a female researcher in health psychology) independently coded three transcripts (phase 2), sorted codes into themes (phase 3), and allocated those to the domains of the TDF (phase 4). For the top-3 barriers and facilitators, the first and last author independently categorized all barriers and facilitators into the TDF domains. In each phase, the researchers frequently discussed the identified codes, themes, and allocation of themes to the TDF domains, to resolve any inconsistencies and coding problems and revise the generated themes. This was done to minimize interpretive bias due to prior understandings of the phenomena under study. Also, the full manuscript was revised by all other authors. Finally, all study participants were sent a copy of a summary report and were invited to provide feedback, which was provided by one participant and incorporated in the results. Quotations were translated from Dutch to English for publication purposes. For patient quotations, patients’ disease statuses were indicated by their CKD stages, including a “T” to indicate kidney transplant recipients (e.g., CKD stage 3 T).

## Results

### Sample characteristics

The final sample consisted of four focus groups with patients (*n* = 24) and four with health professionals (*n* = 23). Each focus group involved four to seven participants. As shown in Tables 1 and B1 (Additional File 3), the patients had a mean age of 62.2 years (range 35.8–85.0 years) and the majority (75.0%) were male. About half of the patients had a kidney function (estimated glomerular filtration rate) of < 30 ml/min per 1.73 m^2^, and almost half had received a kidney transplant. The health professionals had a mean age of 48.4 years (range 25.3–62.7 years) and the majority (73.9%) were female. In each of the focus groups with health professionals, at least one nephrologist, dietician, nurse practitioner, and social worker were present.

**Table 1:** Sample Characteristics of Patients with CKD (*n* = 24) and Health Professionals (*n* = 23) per Focus Group

Participants	No. of Participants	Age Range	Education Level		Gender		CKD stage range	Kidney Transplantation	
			Low	High	Male	Female		Yes	No
Patients	6	55–85	4	1^a^	4	2	4–5	1	5
Patients	7	35–74	4	3	5	2	2–4	7	0
Patients	7	37–79	4	3	6	1	1–5	0	7
Patients	4	48–69	1	3	3	1	2–4	3	1
Professionals	6	42–62	0	6	1	5			
Professionals	6	25–61	0	6	1	5			
Professionals	5	35–57	0	5	2	3			
Professionals	6	34–61	1	5	2	4			

^a^One patient did not complete this question. Abbreviations: CKD, chronic kidney disease; No., number. Low education includes primary, pre-vocational and vocational education; high education includes advanced levels of secondary and tertiary education

### Barriers and facilitators

A summary of the results can be found in Table 2, including the main barriers and facilitators that were brought forward, structured into the 14 overarching TDF domains and 3 COM-B components. Also, the similarities and differences between themes discussed by patients and by health professionals are shown.

**Table 2:** Main Themes (Barriers/Facilitators for Lifestyle Adherence) from Focus Groups, Structured into TDF and COM-B

COM-B component (definition)	TDF Domain (definition)	Themes patients	Themes health professionals
**Capability** (Patients’ psychological and physical capacity to engage in a healthy lifestyle)	**Knowledge** (An awareness of the existence of something)	Knowledge of healthy lifestyle	Knowledge of healthy lifestyle; Beliefs about healthy lifestyle; Beliefs about financial burden
	**Memory, Attention and Decision Processes** (The ability to retain information, focus selectively on aspects of the environment and choose between two or more alternatives)	-	Confusion due to information overload
	**Skills** (An ability or proficiency acquired through practice)	Creativity; Coping with temptations/social pressure	Creativity; Assertiveness
	**Behavioral Regulation** (Anything aimed at managing or changing objectively observed or measured actions)	Breaking habits; Creating routines	Breaking habits; Creating routines
**Opportunity** (All factors external to patients that encourage or discourage healthy lifestyle behaviors)	**Environmental Context and Resources** (Any circumstance of a person’s situation or environment that discourages or encourages the development of skills and abilities, independence, social competence, and adaptive behavior)	Disease characteristics; Material support tools; Characteristics of health care system; Societal characteristics	Disease characteristics; Material support tools; Characteristics of health care system; Societal characteristics; Competing tasks; Psychiatric or cognitive problems
	**Social Influences** (Those interpersonal processes that can cause individuals to change their thoughts, feelings, or behaviors)	Instrumental/emotional support by social environment; Peer pressure	Instrumental/emotional support by social environment; Peer pressure; Professional support
**Motivation** (Patients’ reflective and automatic brain processes that energize and direct behavior, such as habitual processes, emotional responding, and analytical decision-making)	**(Social/Professional) Role & Identity** (A coherent set of behaviors and displayed personal qualities of an individual in a social or work setting)	-	Obedience; Conscientiousness
	**Beliefs about Capabilities** (Acceptance of the truth, reality, or validity about an ability, talent, or facility that a person can put to constructive use)	-	Locus of control; Self-efficacy
	**Optimism** (The confidence that things will happen for the best or that desired goals will be attained)	Focusing at opportunities; Acceptance; Resilience	Focusing at opportunities; Acceptance
	**Emotion** (A complex reaction pattern, involving experiential, behavioral, and physiological elements, by which the individual attempts to deal with a personally significant matter or event)	Depressive feelings; Stress; Anxiety	Depressive feelings; Stress; Anxiety
	**Beliefs about Consequences** (Acceptance of the truth, reality, or validity about outcomes of a behavior in a given situation)	Beliefs about consequences of lifestyle behaviors; Previous experiences with consequences	Beliefs about consequences of lifestyle behaviors; Previous experiences with consequences
	**Reinforcement** (Increasing the probability of a response by arranging a dependent relationship, or contingency, between the response and a given stimulus)	Noticeable effects; Healthy behaviors experienced as punishment	Noticeable effects; Unhealthy behaviors as short-term reward; Punishing unhealthy behaviors
	**Intentions** (A conscious decision to perform a behavior or resolve to act in a certain way)	Intrinsic motivation; Higher-order purposes	Intrinsic motivation; Higher-order purposes
	**Goals** (Mental representations of outcomes or end states that an individual wants to achieve)	Flexibility; Discipline	Goal setting

Parts of this Table are adapted from “Validation of the theoretical domains framework for use in behavior change and implementation research.”, by J. Cane, D. O’Connor, and S. Michie, 2012, *Implementation Science, 7 *[37], p. 13–15. Copyright 2012 by Cane et al. Adapted with permission. TDF: Theoretical Domains Framework; COM-B: Capability, Opportunity, Motivation – Behavior

### Capability

#### Knowledge

Both patients and health professionals emphasized the relevance of patients’ knowledge about lifestyle guidelines. Health professionals reported that patients often have inaccurate beliefs on how to engage in a healthy lifestyle, overestimate its financial burden, and tend to overrate the healthiness of their current lifestyle:

“…they were not at all aware that they took so little steps a day … They were like ‘Oh, I thought I would be closer to those 10.000’.” (Dietician)

In line with this, many patients emphasized the complexity of especially medication prescriptions and dietary restrictions, as well as the need to learn what a healthy lifestyle comprises:

“I would eat half a melon easily. The professor called it ‘wrong fruit’ … with too many sugars and so on. Before I did not know. Ignorance.” (Patient, male, CKD stage 5)

### Memory, attention and decision processes

Health professionals were concerned that patients would get confused by the great amount of often contradictory information, especially for healthy diets and food choices:

“People are really being overwhelmed, they search themselves as well, of course. In a mishmash of information in which they can’t find their way.” (Dietician)

### Skills

Both patients and health professionals emphasized the ability to use creativity in learning new lifestyle behaviors:

“I’m able to cook in such a way that my guests don’t miss salt. I’ve learned many alternatives.” (Patient, female, CKD stage 4)

Health professionals stated that interpersonal skills are required, mainly assertiveness, for instance to ask or search for additional information, to ask for support, and to indicate needs:

“It’s facilitating when a patient asks questions to us as health professionals. So we should train them to ask for what really matters to them.” (Nephrologist)

In line with this, most patients mentioned the importance of the ability to handle social pressure and refuse unhealthy food or cigarettes. Accordingly, some even avoided social gatherings to refrain from unhealthy seductions. Furthermore, some patients made sure that they only had healthy products available at home, as they found it hard to resist temptations:

“When it’s 4 PM, you become tired, and you think ‘screw that apple’, and you open the drawer and think ‘well, what shall I choose?’ What’s available … you grab it more easily.” (Patient, female, CKD stage 2T)

### Behavioral regulation

To automatize healthy lifestyle behaviors, patients and health professionals emphasized consciously breaking habits, creating new routines, and linking new behaviors to existing habits. Health professionals underlined that breaking long-lasting habits is problematic, as it is more comfortable and easy to maintain old habits:

“Then you suddenly have to change something you have been used to doing for 20 years, to lighten a cigarette when you’re a bit stressed.” (Social worker)

Furthermore, disease progress and comorbidities (mainly diabetes) demand patients to frequently adapt their routines of diet and medication intake. Additionally, specifically for medication adherence, deviating from usual routines makes it difficult to remember performing the healthy behavior:

“Sometimes when you go out shopping and it takes longer, then you eat something elsewhere, and then you forget or miss your medication. That takes me by surprise sometimes.” (Patient, male, CKD stage 4)

### Opportunity

#### Environmental context and resources

In all focus groups, characteristics of disease were mentioned to influence patients’ engagement. Disease symptoms, such as fatigue and a lack of energy, make lifestyle adaptations and specifically physical activity difficult. At the same time, some participants stated that a lack of physical symptoms may form a barrier to perceive the urgency to engage in a healthy lifestyle. Health professionals saw this mainly among patients in early, asymptomatic disease stages, but a few transplant recipients also experienced this barrier:

“That’s what makes it [lifestyle adherence] so difficult. I have a kidney function of 18% now, but I do not feel anything [symptoms].” (Patient, male, CKD stage 4T)

A few patients but mainly health professionals pointed out the role of competing tasks, for instance caused by irregularity or busyness at work on top of suffering from a chronic disease. These competing tasks force patients to prioritize, which is often at the expense of a healthy lifestyle, especially of physical activity:

“… and that they [patients] want to use their last bit of energy to work because they really want to maintain that, for instance.” (Nephrologist)

In most focus groups with health professionals, psychiatric illness, addiction, and cognitive decline among patients were believed to hinder a healthy lifestyle, due to insufficient capabilities to change habits, or a lack of insight in their disease and consequences of their behavior.

The use of material resources or tools was mentioned by both patients and health professionals to facilitate lifestyle adherence, such as scales, planners, alarms, medicine boxes, and blood pressure monitors. With regard to the health care system, many health professionals mentioned the short duration of hospital visits, the lack of regular follow-up visits, and the consultation of different health professionals at each visit as barriers to adequately promote their patients’ behavior change in a patient-centered way:

“But if you want to achieve that [behavior change] you need to explain why it is important, … and also check with the patient like ‘how are you going to do that in the upcoming three months, what are the social-context goals you want to achieve’. Yeah then another half an hour has passed, that is impossible.” (Nephrologist)

To overcome these barriers, participants stressed the importance of additional health care support, such as dietetics, physiotherapy, psychotherapy, and social work:

“Nothing is as difficult as behavior change. If you only look at yourself, you can be very motivated, but then to set it in motion and maintain it [is difficult]. You should deploy much more psychotherapists, to make sure that people also get the tools to start behavior change.” (Nurse practitioner)

Regarding barriers from a societal perspective, some participants were critical about the amounts of high-sodium foods in restaurants and supermarkets. Health professionals were concerned about the increase of sedentary behavior in work settings.

### Social influences

In all focus groups with health professionals, the role of their own support was extensively discussed. Many health professionals pointed out the importance of bonding, positive stimulation, regular evaluation, reinforcing progression, and repetition of relevant themes. They all believed that patient-centered care is necessary to achieve that patients engage in a healthy lifestyle. Within patient-centered care, health professionals adapt their communication style and information provision to patients’ personal needs, barriers and facilitators, intellectual abilities, and health literacy, and focus on whatever topic patients find most urgent:

“Sometimes I try to explain sodium but then they mix it up a bit and talk about potassium the whole time, then I think, well then I’ll explain potassium. That is important. To treat what’s on a patient’s mind at that moment.” (Dietician)

Health professionals stressed the importance of emotional and instrumental support by family members, especially regarding smoking and diet. It was believed that living alone and social isolation encourage unhealthy behavior. In addition, they were very concerned about reluctance of family members to participate in patients’ lifestyle behavior change:

“It isn’t only about changing the patients, it’s also about changing their system. You don’t even see their system, that’s even more difficult to change.” (Nephrologist)

Conversely, few patients experienced barriers from their social environment. Some argued that family members do not participate in their lifestyle regimens, or that they are sometimes too pitying or meddlesome. However, most patients were rather positive about family support, especially by partners and children. They indicated that they feel supported when their loved ones think along, keep an eye on their behavior, and participate, especially in healthy eating:

“…my wife always says immediately ‘what’s inside’, in packages and bags … she looks up menus on the internet. … I always say ‘there’s two persons ill, you’re not ill alone’.” (Patient, male, CKD stage 4T)

In almost all patient focus groups as well as by some health professionals, owning a dog and the opportunity to encounter others and engage in social interaction were mentioned to be important facilitators that encourage going out for a walk or bike ride. With regard to social learning and peer pressure, a few health professionals stated that the wish to fit in can be detrimental if patients are surrounded by unhealthy examples of others:

“It is actually ‘not done’ if you say ‘I don’t want to eat that’ at a birthday party, or ‘I’m quitting smoking’ or ‘I won’t visit you tonight because I’ll go for a run’ … you will be judged on that a little. Or you will get comments about it, which won’t make it easier.” (Dietician)

Also, it was mentioned by health professionals that in some cultures, it is common to use a lot of sodium, and it may be impolite to refuse food. Conversely, patients tended to focus on facilitating effects of peer pressure:

“In the past, you fitted in if you smoked, but if you smoke nowadays, you don’t fit in anymore.” (Patient, male, CKD stage 5)

### Motivation

#### (Social/professional) role & identity

Health professionals described patients that succeed in adhering to a healthy lifestyle to be often very obedient, structured, and conscientious:

“The perfectionists they generally achieve more.” (Nephrologist)

“…you just tell them what they have to do, and then they do it exactly. There are also people who behave that way as a part of their personality. … You just say ‘walk 3 times a day’ and they do it.” (Social worker)

#### Beliefs about capabilities

Health professionals emphasized that it is hard to adapt lifestyle habits for patients with an external locus of control. They argued that some patients believe their doctor or partner is responsible for their disease progress or lifestyle, and that they cannot influence their condition themselves:

“When someone says ‘Yes, but my wife cooks’. Then you already know that it will be very hard to get through.” (Dietician)

Similarly, according to some health professionals, patients may lack self-efficacy or have pessimistic beliefs about the complexity of lifestyle behaviors; yet only a few patient participants expressed uncertainty about their own capabilities.

#### Optimism

Participants in all focus groups stressed the importance of optimism, that is, looking at opportunities and alternative possibilities instead of focusing on physical limitations or lifestyle restrictions:

“I see one patient…, he lost two legs in one year. … I thought he would arrive here as a wreck. Well he came in, in that wheel chair, and he said: ‘I’m still able to cook and I love doing that'. The man’s eyes literally beamed.” (Nephrologist)

Patients stressed that their optimism facilitates disease acceptance and resilience. In line with this, both patients and health professionals believed that patients need to accept their condition and lifestyle regimens in order to achieve lifestyle changes:

“Well, what I do, I do not focus on the things which I can’t do anymore, I look at the things I still *can* do.” (Patient, male, CKD stage 4T)

#### Emotion

Some participants recognized that experienced limitations due to CKD and a lack of disease acceptance may contribute to depressive feelings. In most focus groups, the role emotions was already discussed as a barrier for healthy lifestyle behaviors before the quotations from previous studies were shown: Participants agreed that feeling down or depressed forms a barrier to engage in healthy lifestyle behaviors, e.g., physical activity, since depressive feelings are often accompanied by a lack of energy, motivation, persistence, or an inability to see opportunities. Many stated that causes of depressive feelings may also be unrelated to disease, such as work-related problems or an inclination to be pessimistic. A few participants added that chronic disease may make it even more difficult to cope with adversities in other life domains:

“These people already have limitations. Then a setback is even more difficult to handle. More difficult to stay motivated and maintain your lifestyle in order.” (Social worker)

Participants indicated that engaging in healthy behaviors could also be hampered by stress. To deal with stressful situations and to feel less stressed, unhealthy behaviors are being used as coping strategies, especially unhealthy eating and smoking:

“When things happen of which you think, back then I thought it was really true but, that you believe it helps to smoke a cigarette. … especially when things fall short, you ‘need’ some consolation. And you receive that false consolation by a cigarette.” (Patient, female, CKD stage 2T)

Furthermore, participants agreed that anxiety may either hinder or facilitate behavior change. On the one hand, anxiety regarding disease may contribute to an “ostrich policy”:

“Head in the sand, also anxiety, I was very anxious. … I did everything that was unhealthy.” (Patient, male, CKD stage 3T)

On the other hand, concerns about future perspective and especially the occurrence of sudden negative health-related events (e.g., experiencing a heart attack) may cause fear that motivates patients to prevent future complications.

#### Beliefs about consequences

Beliefs about side effects were important barriers for medication-taking behavior. As a facilitator, some participants argued that awareness about the consequences of unhealthy behavior and the effects of a healthy lifestyle may be enhanced by patients’ experiences with dialysis or a kidney transplant themselves or by their relatives:

“My first wife died due to kidney failure, so I know what it is, I saw the entire deterioration process, and that does not make you happy. So you do everything to prevent that from happening.” (Patient, male, CKD stage 2)

“… unless you have something hereditary with grandmother, grandfather, brother, sister who are already in a later stage, then you are more aware of it [the importance of healthy behavior].” (Nurse practitioner)

Specifically, kidney transplant recipients expressed a drive to prevent graft rejection:

“Immunosuppressive drugs, you just take them because the last thing you want is the kidney to be rejected.” (Patient, female, CKD stage 2T)

#### Reinforcement

According to some health professionals, unhealthy behavior, for instance smoking or snacking, provides a short-term reward, while a few patients experienced healthy behavior as some kind of punishment:

“Everything that tastes good, is forbidden.” (Patient, male, CKD stage 5 T).

Most participants reported that noticeable effects, such as visible weight loss, a better physical condition, or a reduction in medication prescriptions, are rewarding and facilitate healthy behavior:

“Results help, either weight loss, or waist circumference diminishes, or you see at such a device at the gym that you got more muscle mass. And [it helps] when you eventually feel it as well. But that small intermediate step of a centimeter, or a kilogram less, or a bit more muscle mass or less fat mass … that helps.” (Dietician)

However, participants stated that it is not always possible to make progress visible or to influence measurement results by adapting lifestyle behavior, due to underlying disease factors:

“Suddenly my body produces a protein while I try so incredibly hard, I follow the dietician’s advice…” (Patient, male, CKD stage 3)

“I think that is very difficult in our patient group … you slowly get worse. You quit smoking, but you don’t get better, you still have difficulties climbing the stairs because your kidney function deteriorates.” (Nurse practitioner)

Some health professionals argued that it helps when unhealthy behavior is punished, for instance by making it more difficult to receive a transplant when a patient does not quit smoking.

#### Intentions

Most participants agreed that a strong intrinsic motivation is needed for patients to engage in behavior change. They stated that in order to succeed, a lifestyle change should be enjoyable and rewarding:

“For example she [social worker] arranged a vegetable garden for me. … In the past I was always active with plants, with the garden. So in that time I had a great experience. That helps to engage in physical activity, growing vegetables yourself, getting to know people. That helped me.” (Patient, male, CKD stage 3T)

Furthermore, participants stated that lifestyle adaptations should be linked to higher-order values and purposes that are personally important for the patients:

“The purpose is not quitting smoking, the purpose is doing fun things with the grandchildren.” (Social worker)

#### Goals

Some patients valued freedom and flexibility in the implementation of lifestyle advices, rather than being too strict:

“One day I do not use salt at all, the other day a little more … that is how I try to do it.” (Patient, female, CKD stage 4)

While health professionals stressed the importance of setting concrete and personally feasible behavioral goals, patients did not mention goal setting specifically. Actually, they stated that discipline should be sufficient to maintain a healthy lifestyle. Some even added that they just want to stay alive and fight for that:

“When you get an advice, you do everything you can to fight for it. You start from the position ‘I want to go on’ and then you try your best.” (Patient, female, CKD stage 5)

#### Ranking of barriers and facilitators

**Fig. 1:**
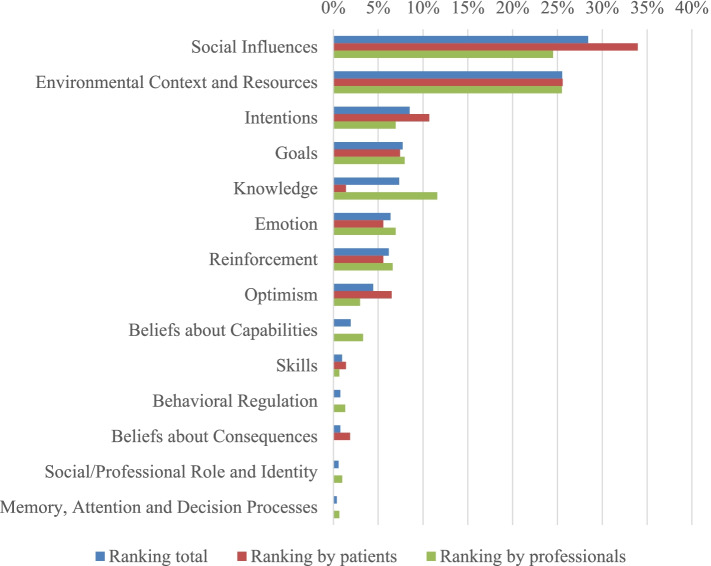
Importance of themes that determine lifestyle adherence in chronic kidney disease ranked by participants. *Note.* Percentages of the points given to themes within each domain of the Theoretical Domains Framework (TDF) are shown, from the total sample and per participant category

Patients as well as health professionals considered barriers and facilitators related to patients’ social and physical environment most important, among which social support by a patient’s partner and others, peer pressure, and physical limitations or complaints. Other themes often ranked by patients were intrinsic motivation, discipline, and an optimistic attitude; whereas health professionals found knowledge about disease and lifestyle, goal setting, disease acceptance, and intrinsic motivation most important. In Fig. 1, a ranking of themes categorized in accordance with the TDF domains is shown.

#### Intervention strategies

Patients suggested that a support program to target the barriers and facilitators should be patient-centered, in order to provide information and support that is tailored to their personal situation and preferences. Additionally, such a program should provide very clear information on what behaviors are healthy, including concrete examples. Especially in one focus group, patients proposed intensive guidance, for instance by a personal coach. Some patients found contact with fellow patients, for example in organized gatherings, useful in order to learn from each other’s experiences, whereas others were hesitant about listening to peers with negative experiences or pessimistic perceptions.

Health professionals had diverse ideas about prerequisites of intervention strategies, such as a positive, empowering approach and repetition of information. Health professionals agreed with patients on a tailored, patient-centered program, and added the importance of small, feasible steps. Some mentioned the utility of psychological intervention strategies for behavior change:

“…if you have a positive self-image, self-esteem, if you are optimistic, happy, then everything will be easier and everything [lifestyle changes] will succeed better. People who are ponderous, not necessarily depressed, are more pessimistic. … Nowadays many initiatives exist, books, internet, coaching, many people have a coach to pay attention to these kinds of things.” (Nephrologist)

## Discussion

With regard to the first aim of this qualitative study, multiple psychosocial barriers and facilitators were revealed across all fourteen domains of the TDF, that may determine the adherence to a healthy lifestyle among patients with CKD not receiving dialysis. Patients and health professionals agreed on the importance of patients’ social and physical environments, intrinsic motivation, and emotional wellbeing. Furthermore, patients stressed discipline and optimism as main determinants, whereas health professionals emphasized knowledge, beliefs about capabilities, and goal setting. As a second research aim, a number of intervention strategies to overcome barriers and promote facilitators were identified in this study. Both patients and health professionals stressed that, since the experienced barriers and facilitators may differ per individual, intervention strategies should be patient-centered and tailored. Tailored interventions could target multiple barriers and facilitators and are personalized to individual needs [24, 25]. Below, the main TDF domains of barriers and facilitators, as well as matching intervention strategies and BCTs that were brought forward by patients and health professionals, will be discussed.

Both patients and health professionals extensively stressed the importance of the degree to which patients’ environments provide the opportunity to engage in a healthy lifestyle. In line with previous studies among CKD populations, this implies that intervention strategies should involve the physical and social environment [26–28]. Health professionals argued that patients’ social environments often hinder engagement in a healthy lifestyle, whereas patients perceived their social network as facilitating. Both perspectives imply the need for BCTs that promote social support [14]. As previously described in research among patients with chronic diseases [29], including patients with chronic kidney disease [30], we found that especially partners and other family members play an integral role in disease management and support. Therefore, it is vital to involve them in behavior change interventions for patients. Possible ways to achieve this suggested by participants included stimulating relatives to join patients’ behavior changes or teaching patients interpersonal skills to indicate their needs and ask for the support they prefer. Also, participants suggested practical environmental resources, i.e., material tools that aid to fit lifestyle adaptations into patients’ personal situation and daily life [14]. For example, planning tools could be used to schedule resting time between physical activities in order to evenly distribute energy levels and diminish fatigue burden.

Regarding motivational barriers and facilitators, participants agreed on the role of a strong intrinsic motivation. This may sometimes be lacking among patients in non-transplant and asymptomatic stages of CKD, who may perceive adherence to lifestyle guidelines to be less urgent. However, enhancing motivation alone is not sufficient to achieve behavior change, as other factors also play an important role herein: Health professionals mentioned in consistence with literature that patients’ capabilities to change their lifestyle may be limited by a lack of knowledge about what a healthy lifestyle comprises [27, 31]. Therefore, a combined approach of BCTs is recommended to enhance both motivation and knowledge [32], which would support patients to put healthy lifestyle behaviors into practice. For instance, in a small intervention study among patients with CKD not receiving dialysis guided by a health psychologist, motivational interviewing techniques to improve intrinsic motivation were combined with education to increase knowledge, tailored to patients’ stages of behavior change [33]. The study showed promising results on medication adherence and emotional wellbeing. The current findings also suggest a need for tailoring behavior change techniques to patients’ stages of behavior change. For example, the BCT of setting personally relevant and feasible goals, which was mainly mentioned by health professionals, may be especially useful to enhance motivation and facilitate the adoption of new behavior in early stages. In contrast, patients stressed the importance of discipline, which should be promoted in later stages to facilitate long-term maintenance of healthy behaviors, for instance by listing and using personal strengths that may aid a patient in persisting (i.e., BCT ‘valued self-identity’; 14).

Regarding emotional wellbeing, patients and health professionals agreed on the negative impact of psychological distress, including symptoms of depression, anxiety, and stress. A link between psychological distress and non-adherence to lifestyle recommendations is not surprising, as psychological distress symptoms are related to reduced levels of energy, motivation, self-efficacy, self-regulatory resources, and social support, which all may form strong barriers for engagement in a healthy lifestyle [7, 11]. Intervention strategies in chronic disease evaluated in published research usually focus either on diminishing psychological distress or on improving lifestyle behaviors [34﻿]. It is desirable to target both in an integrated way, for instance by providing self-management support using cognitive-behavioral therapy specialized for adjustment to chronic disease [17﻿]. In integrated interventions, patients can be stimulated to set goals and engage in behaviors that both diminish psychological distress and improve healthy lifestyle behaviors [7﻿]. For example, not only BCTs could be deployed that directly target lifestyle behaviors, such as enhancing motivation or knowledge, but also that seek to reduce negative and enhance positive emotions to facilitate performance of the desired behaviors, e.g., by cognitive restructuring of negative thoughts and beliefs [14].

This study has several strengths. To our knowledge, this is the first qualitative study in which barriers and facilitators for engaging in a healthy lifestyle were explored across the full range of lifestyle recommendations in CKD care. Since perspectives of patients and health professionals working with different CKD stages were involved, including kidney transplant patients, it can be argued whether the results can be generalized to populations with various disease courses and characteristics (e.g., symptomatic or not). On the one hand, some of the barriers and facilitators found were rather specific for the CKD population, such as those related to the gradual disease progress with asymptomatic early stages and burdensome treatments in severe end stages, including the often complex dietary restrictions and medication prescriptions that vary across stages. Also, some facilitators were specifically relevant for (potential) kidney transplant recipients, such as adherence to healthy lifestyle behaviors driven by a motivation to enhance their eligibility for transplantation or to prevent graft rejection. These disease-specific themes should be taken into account when supporting patients with CKD not receiving dialysis. On the other hand, patients with CKD often suffer from comorbidities (e.g., type 2 diabetes mellitus) and many themes, such as psychological distress, did not seem disease-specific and have been found applicable to other chronically ill populations for which engaging in healthy lifestyle behavior is also essential, such as for patients with diabetes or cardiovascular diseases [35–37]. Furthermore, participants in our study have different education levels, suggesting that the findings may also be applicable to under-served groups with low education levels and possibly low health literacy, since education level has been found predictive of health literacy [38]. As we did not measure health literacy or other chronic conditions in the current study, additional research could further investigate generalization to different populations and health behaviors.

Some limitations should be taken into account. As a possible source of bias in data collection, one could argue that the relevance of psychological distress may have been overestimated due to the quotations shown and the specific question about this theme. However, in most focus groups, the importance of psychological distress was already discussed by participants as a consequence of living with CKD and as a barrier for healthy lifestyle behaviors before the quotations and specific question were used. Furthermore, adherence to a healthy diet was more prevalently discussed than the other relevant lifestyle behaviors. This result may indicate that dietary adherence is the most important and complex lifestyle behavior in this population. However, the frequent discussion of dietary adherence may also be explained by the presence of a dietician in each focus group among health professionals (whereas in only one focus group, a physiotherapist participated), and the fact that dietetics is the only specialized hospital lifestyle support that is common in Dutch CKD care. Regarding data analysis, the inductively created codes fitted the TDF well and a main advantage of using this meta behavior change framework is its systematic synthetization of a large amount of behavior change theories, which taps into the challenges of translating theories into practice and minimizes the risk of missing relevant constructs [29, 39]. However, some challenges were experienced when mapping the data onto the TDF. Foremost, not all domains are mutually exclusive and mainly the ‘environmental context and resources’ domain seemed rather broad and not very well conceptualized [40]. Therefore, this domain was experienced to be a receptacle of a wide range of themes and thus could seem over-represented. Furthermore, the TDF was originally developed for health professionals’ behavior [41], and difficulties were experienced when translating some domains to patients’ behavior, for instance with ‘social/professional role & identity’. These challenges were solved by comparing our categorizations to other studies focused on kidney patient disease management behaviors that used the TDF [16, 23]. Additionally, we applied constant researcher triangulation and discussion, in order to reach interpretation consistency and a satisfactory inter-rater reliability [20, 21﻿].

The results, which show a great variety of psychosocial barriers and facilitators that may differ per individual patient, imply that tailored psychosocial intervention strategies could be a promising approach to support patients with CKD in lifestyle behavior change. To be able to support patients in their personal needs, first, psychosocial and lifestyle-related difficulties of an individual patient should be detected. However, it may be difficult to assess psychosocial barriers that are not readily observable and patients may hesitate to disclose personal information in a routine hospital visit [42, 43]. Among patients in our focus groups, little discussion of support by their health care providers took place, which may indeed indicate a gap of discussing personal barriers and facilitators for healthy lifestyle behaviors in patient-provider communication. This lack of discussion may lead to misunderstandings and discrepancies between patient needs and actual support from health professionals, especially since our findings show that determinants perceived as important by health professionals, such as knowledge, do not always align with determinants that matter to patients themselves. In addition, health professionals mentioned the low frequency and duration of contact moments as a barrier to adapt their aid to their patients’ needs. A suitable first step in tailored intervention design may therefore be a screening instrument including the main TDF domains by which patients could indicate personal barriers and facilitators. Such a tool could aid health professionals to accurately and efficiently address potential barriers in consults, adjust lifestyle advices to their patients’ situation, monitor and reinforce progress, and refer patients to more specialized support or treatment when needed [17, 44]. As a second step, such a specialized and tailored treatment could be developed by translating the TDF domains to intervention strategies and BCTs that fit the target behavior and population best [14]. To guide these steps, the Behavior Change Wheel, a framework for intervention design, could be used [14]. As an example, we refer to the steps taken in the development of the E-GOAL eHealth care pathway, described elsewhere [45﻿], in which we matched the insights of the current focus group study to intervention content. For instance, to promote the TDF domain reinforcement, we included the BCTs ‘self-reward’ and ‘material rewards’, in an exercise to select a personally relevant contingent reward to reinforce progress in the desired behavior change. Noteworthy, many barriers, facilitators, and intervention strategies identified in this study were found valuable for multiple lifestyle behaviors relevant in CKD management, and previous research proposed that the interrelatedness of different lifestyle behaviors warrants an integrated approach [27﻿]. This suggests that a single intervention could be adapted to the lifestyle behavior of patients’ preference. Development, evaluation, and implementation of such an integrated intervention should preferably take place in a research setting in close collaboration with patients, health professionals, and other stakeholders in clinical practice, to investigate its feasibility, acceptability, cost-effectiveness, and effectiveness in improving patients’ lifestyle and health outcomes.

## Conclusions

This focus group study presents a broad range of barriers and facilitators that determine engagement in healthy lifestyle behaviors among patients with CKD not receiving dialysis. The great amount of mainly environmental, motivational, and emotional barriers experienced by patients, may explain why many of them do not succeed in adhering to the CKD lifestyle recommendations. Participants in this study stressed the impact of psychosocial barriers and facilitators for lifestyle adherence, such as psychological distress, which may have been somewhat overlooked in previous research. Furthermore, the current study identified intervention strategies to overcome barriers and promote facilitators, as the TDF domains can be translated to matching BCTs. Developing and investigating interventions that address psychosocial barriers and facilitators, and that combine screening and treatment, allows for better tailoring to patients’ needs, by identifying and treating individually experienced psychosocial barriers for adherence to lifestyle guidelines.

## Supplementary Information

**Additional file 1.**

**Additional file 2.**

**Additional file 3.**

**Additional file 4.**

## Data Availability

Transcripts of focus groups are stored in electronic format on secure servers at the Leiden University. All data generated during analysis in the study are also stored on these servers; where considered necessary for publication, they have been included in this article. All other data that support the findings of this study are available on request from the corresponding author (C.C.). The data are not publicly available due to them containing information that could compromise research participants’ privacy/consent.

## Abbreviations

CKD: Chronic kidney disease
TDF: Theoretical Domains Framework
BCT: Behavior change technique
E-GOAL: E-health Guidance in identifying and Overcoming psychological barriers for Adopting a healthy Lifestyle among patients with chronic kidney disease
COREQ: COnsolidated criteria for REporting Qualitative research
COM-B system: Capability, Opportunity, and Motivation-Behavior system
