# Supplementary material for: Psychosocial barriers and facilitators for adherence to a healthy lifestyle among patients with chronic kidney disease: a focus group study
Source: BMC Nephrol. 2022 Jun 11;23:205. doi: 10.1186/s12882-022-02837-0 (PMC9188106; doi:10.1186/s12882-022-02837-0)
Supplement: Supplementary file 2 — Additional file 2. [file 12882_2022_2837_MOESM2_ESM.docx]

**Additional File 2**

**Question Guide of the Focus Groups**

| Table A1. Question Guide of the Focus Groups. |
| --- |
| 1. What are the most important lifestyle adaptations you made or think you should make? *(only in patient focus groups)* |
| 2. What do you perceive as the most important consequences of (your) kidney disease? |
| 3. Facilitators: what helps or has helped you/your patients to engage in a healthy lifestyle? |
| 4. Barriers: what makes or has made it difficult for you/your patients to engage in a healthy lifestyle? |
| 5. What barriers and facilitators are specific to a healthy diet/a healthy weight/physical activity/no smoking/medication adherence? |
| 6. We think that psychological factors may play a role. *Example quotations shown:*  *“I think a lot of the time that’s what can make the whole self-management thing so difficult to stick to, it’s the thing of wanting to be your old self.”*^a^  “*…these little conditions, they stop you doing things, and then your motivation, if you’re feeling down and you’re depressed, then your motivation’s not there.*”^b^  Do you recognize this? Why? |
| 7. If you would have the opportunity to design your own program to support patients with CKD in targeting the mentioned barriers and facilitators, what should be included in such a program? |

^a^(Gordon et al., 2017, p.e212); ^b^Coventry, Fisher, Kenning, Bee, & Bower, 2014, p. 7)
