# Supplementary material for: Psychosocial barriers and facilitators for adherence to a healthy lifestyle among patients with chronic kidney disease: a focus group study
Source: BMC Nephrol. 2022 Jun 11;23:205. doi: 10.1186/s12882-022-02837-0 (PMC9188106; doi:10.1186/s12882-022-02837-0)
Supplement: Supplementary file 3 — Additional file 3. [file 12882_2022_2837_MOESM3_ESM.docx]

**Additional File 3**

**Sample Characteristics**

| Table B1. Sample Characteristics of Patients with CKD (*n* = 24) and Health Professionals (*n* = 23). | | | | | | |
| --- | --- | --- | --- | --- | --- | --- |
| Characteristics | Patients | | Professionals | | | |
| Mean age (SD) | 62.2 | (13.7) | | 48.4 | (11.5) |  |
| Male gender *n* (%) | 18 | (75.0) | | 6 | (26.1) |  |
| Country of birth Netherlands *n* (%) | 20 | (83.3) | | 21 | (91.3) |  |
| Married/partnered *n* (%) | 20 | (83.3) | | 20 | (87.0) |  |
| Having children *n* (%) | 20 | (83.3) | | 13 | (56.5) |  |
| Level of education *n* (%)^a^ |  |  | |  |  |  |
| Low (primary, pre-vocational and vocational) | 13 | (56.5) | | 1 | (4.3) |  |
| High (advanced secondary and tertiary) | 10 | (43.5) | | 22 | (95.7) |  |
| Work status *n* (%)^a^ |  |  | |  |  |  |
| Full-time | 3 | (13.0) | | 12 | (52.2) |  |
| Part-time | 3 | (13.0) | | 11 | (47.8) |  |
| Voluntary work | 5 | (21.7) | |  |  |  |
| School/studies | 1 | (4.3) | |  |  |  |
| Home/retired | 8 | (34.8) | |  |  |  |
| Disabled due to health | 3 | (13.0) | |  |  |  |
| Kidney transplantation *n* (%) | 11 | (45.8) | |  |  |  |
| CKD stage *n* (%) |  |  | |  |  |  |
| 1 | 1 | (4.2) | |  |  |  |
| 2 | 4 | (16.7) | |  |  |  |
| 3 | 8 | (33.3) | |  |  |  |
| 4 | 6 | (25.0) | |  |  |  |
| 5 | 5 | (20.8) | |  |  |  |
| Mean years in CKD treatment (SD) | 11.6 | (8.1) | |  |  |  |
| Health care profession *n* (%) |  |  | |  |  |  |
| Internist-nephrologist |  |  | | 7 | (30.4) |  |
| Dietician |  |  | | 6 | (26.1) |  |
| Social worker |  |  | | 4 | (17.4) |  |
| Nurse practitioner |  |  | | 4 | (17.4) |  |
| Nurse |  |  | | 1 | (4.3) |  |
| Physiotherapist |  |  | | 1 | (4.3) |  |

^a^One patient did not complete this question. Abbreviations: CKD, chronic kidney disease.
