# Supplementary material for: Psychosocial barriers and facilitators for adherence to a healthy lifestyle among patients with chronic kidney disease: a focus group study
Source: BMC Nephrol. 2022 Jun 11;23:205. doi: 10.1186/s12882-022-02837-0 (PMC9188106; doi:10.1186/s12882-022-02837-0)
Supplement: Supplementary file 4 — Additional file 4. [file 12882_2022_2837_MOESM4_ESM.docx]

**Additional File 4**

**E-GOAL Study Group**

Dr. Sandra van Dijk, Leiden University

Prof. Dr. Andrea W. M. Evers, Leiden University

Dr. Henriët van Middendorp, Leiden University

Dr. Paul van der Boog, Leiden University Medical Center

Prof. Dr. Gerjan Navis, University Medical Center Groningen

Prof. Dr. Luuk B. Hilbrands, Radboud university medical center

Dr. Yvo W. J. Sijpkens, Haaglanden Medical Center

Dr. Yvette Meuleman, Leiden University Medical Center

Drs. Karin Boslooper-Meulenbelt, University Medical Center Groningen

Dr. Sasja Huisman, Leiden University Medical Center

Jan Luijten, patient research partner

Carla van Dorp, patient research partner

Prof. dr. Friedo W. Dekker, Leiden University Medical Center

Dr. Joris I. Rotmans, Leiden University Medical Center

Prof. Dr. Ton J. Rabelink, Leiden University Medical Center

Prof. Dr. Niels H. Chavannes, Leiden University Medical Center

Dr. J. K. Sont, Leiden University Medical Center

Dr. E. M. Scholten, Haaglanden Medical Center

Drs. Katja Cardol, Leiden University
